# Anti-Obesity Effects of Chitosan and Its Derivatives

**DOI:** 10.3390/polym15193967

**Published:** 2023-10-01

**Authors:** Balzhima Shagdarova, Mariya Konovalova, Valery Varlamov, Elena Svirshchevskaya

**Affiliations:** 1 Research Center of Biotechnology, Russian Academy of Sciences, 119071 Moscow, Russia; varlamov@biengi.ac.ru; 2 Shemyakin-Ovchinnikov Institute of Bioorganic Chemistry of the Russian Academy of Sciences, 117997 Moscow, Russia; mariyavkonovalova@gmail.com

**Keywords:** obesity, chitooligosaccharides, chitosan, chitosan derivatives, anti-obesity

## Abstract

The number of obese people in the world is rising, leading to an increase in the prevalence of type 2 diabetes and other metabolic disorders. The search for medications including natural compounds for the prevention of obesity is an urgent task. Chitosan polysaccharide obtained through the deacetylation of chitin, and its derivatives, including short-chain oligosaccharides (COS), have hypolipidemic, anti-inflammatory, anti-diabetic, and antioxidant properties. Chemical modifications of chitosan can produce derivatives with increased solubility under neutral conditions, making them potential therapeutic substances for use in the treatment of metabolic disorders. Multiple studies both in animals and clinical trials have demonstrated that chitosan improves the gut microbiota, restores intestinal barrier dysfunction, and regulates thermogenesis and lipid metabolism. However, the effect of chitosan is rather mild, especially if used for a short periods, and is mostly independent of chitosan’s physical characteristics. We hypothesized that the major mechanism of chitosan’s anti-obesity effect is its flocculant properties, enabling it to collect the chyme in the gastrointestinal tract and facilitating the removal of extra food. This review summarizes the results of the use of COS, chitosan, and its derivatives in obesity control in terms of pathways of action and structural activity.

## 1. Introduction

Chitosan is a deacetylated derivative of the natural polymer chitin. It is a linear polysaccharide consisting of β-(1-4)-linked D-glucosamine and N-acetyl-D-glucosamine links. Chitosan is biocompatible, biodegradable, and has low toxicity [1]. Due to its polycationic nature and the presence of hydroxyl groups in its structure, it can form ionic and hydrogen bonds. The presence of reactive amino and hydroxyl groups facilitates chemical modification of the chitosan molecule [2]. Any change in the structure of chitosan leads to the modification of its physicochemical properties such as solubility, charge, and hydrophobicity/hydrophilicity. This change in biological properties can increase its antibacterial, antiviral, or antitumor activity [3]. Chitosan nanoparticles are used as drug delivery systems; chitosan gels promote accelerated skin wound regeneration; and the powder form acts as a hemostatic and antimicrobial agent [4,5,6].

Obesity is one of the most serious health problems of our time. The number of people suffering from obesity is growing every year. The WHO estimates that it affects more than a billion people worldwide, and by 2025, about 167 million people will be unhealthy due to obesity, leading to a range of health problems of the cardiovascular system, liver, kidneys, joints, and reproductive system. It also increases the number of people with type 2 diabetes, as well as cancer and mental illness. During the COVID-19 pandemic, obese people were three times more likely to be hospitalized [7].

Obesity is a multifactorial disease characterized by increased fat deposition [8]. Few cases of obesity are due to monogenic causes, and there is an increasing number of cases of obesity due to environmental factors and individual genetic predisposition [9]. Changes in the gut microbiome, infectious processes, medication side effects, sleep disturbances, maternal age at birth, duration of breastfeeding, negative environmental factors, and epigenetic changes contribute to the development of obesity [10,11].

Over the past two decades, several strategies have been used to identify the genetic determinants of obesity. These include severe obesity studies, genome-wide linkage studies (GWLS), genome-wide association studies (GWAS), and candidate gene analyses [12]. Among the GWAS results, the first obesity-prone locus to be identified was the fat mass obesity-associated (FTO) gene. This gene has by far the largest effect on the risk of obesity phenotypes. Each FTO risk allele is associated with a 20–30% increased risk of obesity and a 1–1.5 kg increase in body weight [13]. GWAS studies have identified 127 new loci associated with common forms of obesity in diverse populations of different ethnicities and ages [12].

Most cases of obesity in developed countries are a result of an excess and easy access to unhealthy food [8]. Eating behavior is regulated by the hypothalamic central nervous system where signaling molecules such as leptin, insulin, neuropeptide Y (NPY), agouti-related protein, proopiomelanocortin, and adiponectin play important roles. The central nervous melanocortin system is a key point for nutrient-sensitive neural networks that control appetite and metabolic responses [14]. This system plays an important role in the regulation of energy balance and homeostasis processes. An imbalance between energy intake and energy expenditure leads to obesity [8].

Modern medical treatment of obesity involves the use of FDA-approved drugs that have different mechanisms of action. Among them, orlistat reduces the absorption of dietary fats; phentermine/topiramate, lorcaserin, naltrexone/bupropion, and liraglutide all act centrally, suppressing appetite [15,16]. The most popular drug, semaglutide, a glucagon-like peptide-1 receptor agonist, was developed to treat diabetes [17]. Semaglutide increases the production of insulin. Severe cases can be corrected via surgical interventions such as gastric banding with laparoscopy, or biliopancreatic diversion with duodenal switching. In addition, devices such as an intragastric balloon or endoscopic sleeve gastroplasty can be used during surgical intervention [18]. Preventive, non-pharmacologic treatments of obesity include changes in eating habits and an increase in physical activity. It is essential that everyone is educated in good habits, and has access to and can afford a healthy diet. Effective steps include imposing taxes on “junk food” such as sugary drinks and providing greater access to affordable healthy foods. Safe walking, cycling, and recreational spaces should be created where people live [7].

Clinical and epidemiological studies have shown that metabolic syndrome begins with central obesity, where adipose tissue plays a special role in the development of the metabolic syndrome [19]. Adipocytes are sensitive to insulin, which is one of the main regulators of metabolism. The overdevelopment of adipose tissue as a result of the hyperplasia and/or hypertrophy of adipocytes leads first to the development of obesity, and then, to insulin resistance [20]. From an evolutionary point of view, adipose tissue is necessary for the storage of energetically valuable molecules such as triglycerides. Adipose tissue secretes hormones called adipokines. The most studied are leptin and adiponectin. Leptin regulates the synthesis and release of the hunger-mediator peptide NPY, thereby inducing a feeling of fullness. Adiponectin stimulates the β-oxidation of fatty acids and maintains blood glucose level [21].

## 2. Effects of Chitooligosaccharides and Chitosan against Obesity

### 2.1. Chitooligosaccharides (COS)

Chitosan derivatives with MW < 10 kDa are considered COS, which consist of β-(1-4)-linked D-glucosamine with a deacetylation degree (DD) greater than 90% and a polymerization degree less than 20, obtained through the deacetylation and depolymerization of chitosan or chitin [22,23]. Compared to chitosan, COS have much higher solubility and lower viscosity under physiological conditions due to their shorter chain length and higher DD [23]. COS is readily absorbed through the intestinal epithelium and is preferentially distributed in the liver, spleen, and kidney. It is mainly excreted with urine. Interestingly, COS with a degree of polymerization >6 have greater biological activity, including antimicrobial, antitumor, and immunostimulatory activity, compared to smaller COS [23]. Chitosan oligomers interact with some proteins in the body, causing anti-obesity and anti-diabetic effects such as the inhibition of the intestinal enzymes involved in fat and glucose absorption, the inhibition of PPAR-γ, the stimulation of glucokinase, and the suppression of phosphoenolpyruvate carboxylase in the liver [22,24].

Multiple in vitro studies have demonstrated that COS (<3 kDa) treatment of the preadipocyte 3T3-L1 decreases PPAR-γ expression and reduces leptin, adiponectin, and resistin levels, preventing adipogenic differentiation [25,26,27,28,29,30,31] (Table 1). In vivo COS, used as a food supplement, decreased body weight, the accumulation of subcutaneous and total fat, and the content of TC, TG, and LDL-C, and improved blood serum HDL-C levels in rats [32] (Table 1).

COS can improve dyslipidemia and prevent weight gain by inhibiting adipocyte differentiation in obese rats. Bahar et al. studied the effect of COS with MW < 1, 1–3, 3–5, and 5–10 kDa on the differentiation of 3T3-L1 cells and determined their antiadipogenic potential. The inhibition of lipid accumulation, free glycerol release, and the expression of adipose-related genes was observed under the action of COS. The listed indicators varied depending on the concentration and MW, with COS 5–10 kDa showing the greatest effect. The inhibition of adipogenesis at all studied COS concentrations was associated with IL-6 and prostaglandin-endoperoxide synthase 2 gene expression. At the same time, IL-6 probably induces the breakdown of lipid molecules and prevents lipid accumulation during adipogenesis [33]. It was also found that COS with MM 5–10 kDa suppressed lipid metabolism gene expression and lipid accumulation during adipocyte differentiation. COS also inhibited the expression of the leptin gene during adipogenesis [25]. The inhibition of leptin is important because leptin is turned on by epigenetic modulation when preadipocytes are stimulated to initiate the process of adipogenesis [36].

Obesity models using experimental animals play an important role in the study of the pathogenesis and therapy of this metabolic disorder. The commonly used model is the high-fat diet (HFD). It is important to use HFDs with a defined macro- and micronutrient composition and a clear description of the diet. The development of obesity in animals using HFD is pathophysiologically very similar to the human disease, allowing for the study and intervention of obesity in the experimental studies [37].

Deng et al. found that high doses of COS (1200 mg/kg) with MW ≤ 1kDa, used for 8 weeks in HFD-induced obese rats, reduced body weight and lessened the accumulation of perirenal, epididymal, subcutaneous, and total fat. The ratio of fat to body weight decreased by 19%, serum TC levels by 37%, and TG levels by 50% in the COS group compared to the HFD one. Serum LDL-C levels were significantly reduced and serum HDL-C levels were improved in COS-fed rats [32]. COS used as a food supplement were more effective in weight loss compared to the commercial drug orlistat. Serum total cholesterol and LDL-C levels were significantly reduced in all treatment groups compared with the control HFD one. Various doses of COS reduced the mRNA expression levels of PPAR-γ and liver X receptor alpha mRNA in white adipose tissue [29]. COS significantly enhanced the thermogenic ability of HFD-induced obese rats and increased the expression of brown adipose tissue genes and proteins such as uncoupling protein 1 (UCP1), PPAR coactivator-1α, PR/SET domain 16 (PRMD16), and activating transcription factor 2 (ATF2), in white adipose tissue (WAT) and brown adipose tissue (BAT). By studying the function and mechanism of thermogenesis, it has been shown that COS can increase brown WAT and BAT thermogenesis to suppress obesity [34].

The anti-obesity properties of COS were also investigated in ob/ob mice. An increase in body weight (12%) and a decrease in food intake (13%) and in lipid levels (29%) were observed with COS supplementation. An improvement in plasma adiponectin and resistin levels and the suppression of adipose tissue-specific TNF-α and IL-6 gene expression was shown. The expression of PPAR-g genes confirmed enhanced adipokine production [30]. In addition, the anti-obesity effect of COS capsules (MN ≤ 1000 g/mol; DD 95.6%) was investigated in rats suffering from HFD-induced obesity. The administration of COS for 8 weeks resulted in the activation of the JAK2-STAT3 signaling pathway to attenuate leptin resistance. COS can not only decrease body weight gain, but can also correct serum alanine aminotransferase and aspartate aminotransferase levels. Therefore, COS may be a potentially valuable natural product for the prevention and treatment of obesity [35].

However, a toxic effect of COS was reported by Chiu et al. [38]. Diets supplemented with 5% COS caused liver damage in animals on the HFD. Liu et al. studied COS toxicity (0.7kDa, DD 100%) using non-obese rats. The results showed that the addition of 5% COS to the diet of mice for 12 weeks did not cause lipid metabolism disturbance and hepatotoxicity in normal rats [39]. To avoid side effects, it is necessary to study chitosan toxicity in more detail.

### 2.2. Chitosan

The biological properties of chitosan with MW > 10 kDa and its derivatives are highly dependent on the MW and DD of the polymer [22]. Depending on the aim, chitosan preparations with different properties should be selected. Multiple data discussed above demonstrated that COS are effective both in vitro and in vivo. At the same time, the international market has a number of dietary supplements with chitosan designed for the treatment of obesity, hypercholesterolemia, and hypertension [40,41]. Commercial supplements rarely describe the MW of chitosan. Still, it is shown that any MW chitosan used as a food supplement reduces the absorption of fat and cholesterol [42]. Chitosan diets generally reduce plasma lipid levels and increase the excretion of fat and cholesterol in the feces [31].

Sumiyoshi and Kimura investigated the effects of chitosans with MW 21, 46, and 130 kDa in mice by using HFD for 20 weeks (Table 2). Chitosan with MW 46 kDa was the most effective; its lipid-lowering effect was probably due to an increase in the fecal excretion of fat and/or bile acids as a result of bile acid binding, and a decrease in the absorption of dietary lipids (triacylglycerol and cholesterol) from the small intestine as a result of inhibition of pancreatic lipase activity. MW 46 kDa chitosan did not cause liver or renal damage [43]. Supplementation with chitosan MW 80 kDa, DD 84%, at a concentration of 0.5% to rats receiving HFD for 10 weeks, showed that it resulted in body weight loss and liver and adipose tissue fat decreases in the rat model. It also corrected the imbalance of lipid profiles in the plasma, liver, and feces, as well as the activity of intestinal disaccharidases in the HFD rat model. A significant decrease in the activity of mucinase and β-glucuronidase in the fecal microflora was also observed [44].

The analysis of chitosan with MW 300 kDa, DD 72%, in vitro using 3T3-L1 fibroblasts demonstrated increased platelet-derived growth factor-induced mitochondria fragmentation, and enhanced cell proliferation and migration [46]. Another work showed that the oral administration of chitosan MW 300 kDa, DD 97%, to mice for 5 weeks resulted in a 9.3% weight loss. Chitosan reduced epididymal fat pad and intra-abdominal fat thickness by 10%, plasma TC levels by 26%, TG by 40%, and LDL-C by 38%, with no significant effect on plasma HDL-C levels [31]. Chiu et al. studied the change in cholesterol levels in rats treated with HFD when chitosans with different molecular masses of 740, 80, and 0.7 kDa were supplemented for 8 weeks. Chitosan 80 and 740 kDa significantly decreased liver total cholesterol levels, increased TC levels in feces, suppressed the expression of the PPARα gene, and effectively reversed the HFD-inhibited/induced expression of low-density lipoprotein receptor (LDLR) and cholesterol-7α-hydroxylase (CYP7A1) genes. Both low- and high-molecular-weight chitosan effectively reduced hypercholesterolemia and regulated cholesterol homeostasis [38]. Furthermore, chitosan accelerated brown fat formation and thermogenesis by increasing the expression of UCP1, PRDM16, and PGC-1α genes, thereby accelerating energy metabolism and increasing fat consumption in HFD-induced obese rats. Therefore, chitosan with MW > 10 kDa also can potentially be used for obesity prevention and treatment [34].

Taken collectively, chitosans with different MWs (10–700 kDa) all have close activity, mediated mostly by binding lipids in food stimulating their excretion with feces.

## 3. Effect of Chitosan Derivatives on Obesity

The chemical modification of chitosan is possibly due to the presence of reactive amino groups at the C-2 atom and primary and secondary hydroxyl groups at the C-3 and C-6 atoms in the chitosan molecule. This approach makes it possible to change the chemical properties and enhance or add biological activity depending on the nature of the introduced groups, while preserving the natural backbone of chitosan [47]. To improve water solubility, pH sensitivity, and the targeting of chitosan derivatives, chitosan is often modified via acylation, carboxylation, alkylation, and quaternization [48]. Modified chitosans are most commonly used in the field of biomedicine, such as drug delivery, antitumor agents, the development of wound dressings, and promoting bone and tissue regeneration [49].

The results of chitosan derivatives’ anti-obesity activity are shown in Table 3. Phosphorylated glucosamine (glucosamine-6-phosphate, PGlc) decreased lipid accumulation and suppressed PPAR-γ, sterol regulatory element-binding protein 1 (SREBP1), and protein C/EBP-α expression in 3T3-L1 cells. In addition, PGlc induced a significant increase in preadipocyte factor 1 expression and the suppression of gene promoters such as adipocyte fatty acid-binding protein, fatty acid synthase lipoprotein lipase, and leptin [27].

The sulfation of chitosan is primarily necessary to impart anticoagulative properties upon the polymer, but in addition, such derivatives are characterized by antioxidant, antibacterial, and antiviral properties [47]. (N,O)-sulfated chitosan was also effective in reducing lipid accumulation and triglyceride levels, and facilitated lipolysis in differentiating 3T3-L1 preadipocyte cells. In addition, the gene expression and protein levels ofPPAR-γ and C/EBP-α were significantly reduced [28].

O-Carboxymethyl chitosan (O-CMC) and quaternized chitosan (N-[(2-hydroxy-3-N,N-dimethylhexadecylammonium)propyl]chitosan chloride, N-CQC) are some important chitosan derivatives that have different charges and, consequently, different properties [52]. Both O-CMC and N-CQC demonstrated anti-obesity effects not only in vitro in 3T3-L1 cells but also in vivo in a rat HFD model. O-CMCs and N-CQCs decreased plasma leptin, glucose, insulin, and total cholesterol levels, suppressed leptin and resistin mRNA expression, and increased adiponectin and PPAR-γ mRNA expression [50].

Li et al. synthesized chitosan derivatives containing coumarins to enhance the antioxidant activity of chitosan. No cytotoxicity was observed in 3T3-L1 cells when incubated with chitosan and its derivatives at all studied concentrations from 1 to 1000 μM. Meanwhile, the synthesized chitosan derivatives stimulated the growth of 3T3-L1 cells, which could be attributed to their high antioxidant activity [53]; however, this could be a result of the anti-differentiation effect.

Thus COS, high-MW chitosans, and their derivatives all demonstrate close properties and can be used as anti-obesity agents. Due to the high variability in chitosan samples it is difficult to conclude which preparation demonstrates the highest anti-obesity effects. Our hypothesis is that the major anti-obesity mechanisms of chitosan are due to its flocculant effect in the gut. Chitosan, due to its charge and independently of its weight, DD, or modification, absorbs and increases the chyme volume, stimulating its movement in the intestine.

## 4. Regulation of Gut Flora by Chitosan and COS

Chitosan and COS may find uses as dietary supplements that can regulate intestinal flora. The human gut microbiota consists of 10^13^–10^14^ microorganisms [54]. The commensals can be affected by numerous factors such as different environmental conditions, diet, antibiotics, or physical activity [55]. The gut microbiota directly affects nutrient absorption as well as fat accumulation in the human intestine [56]. Disturbances in the gut microbiota are closely related to the pathogenesis of obesity and related metabolic syndromes [57].

The prolonged use of chitosan can affect the gut microbiome. A chitosan-supplemented diet decreased the number of the obesity-related species *Coprobacillus cateniformis* and *Clostridium leptum.* Their numbers correlate with increased serum leptin levels. Contrary to this, *Clostridium lactatifermentans* and *Clostridium cocleatum*, the numbers of which positively correlate with the serum lipid levels, were significantly reduced [58]. He et al. used LMW-COS-H and LMW-COS-L, with MWs of 0.9 kDa and 0.36 kDa, respectively. Compared with LMW-COS-L, LMW-COS-H was more effective in improving metabolic disturbances induced by HFD. The addition of LMW-COS to the diet induced overall changes in the gut microbiota, which were significantly associated with metabolic parameters. LMW-COS both significantly increased the abundance of beneficial gut bacteria such as *Akkermansia* and *Gammaproteobacteria* and decreased the relative abundance of inflammatory bacteria such as *Erysipelatoclostridium* and *Alistipes*. Thus, it is possible that LMW-COS act as a prebiotic that regulates dysfunctional gut microbiota, alleviate low-grade inflammation, and maintain the intestinal epithelial barrier [57].

Elebeedy et al. used chitosan as a prebiotic alone or in combination with a probiotic for 45 days in rats. The rat kidneys showed a normal histologic structure of renal tubules when treated with probiotics, chitosan, and their mixture. A decrease in total body weight was observed when these approaches were combined [59].

In addition, chitosan can be used to deliver probiotics. Probiotic bacteria must first be protected from ingestion in the stomach. Microencapsulation is one of the most utilized methods. In a digestion test, chitosan preserved the required amount of viable probiotic bacteria [60]. The coating of microcapsules with chitosan or a combination of chitosan and alginate contributes to the survival of many probiotic bacteria (Bifidobacterium and *Lactobacillus* spp., *L. bulgaricus*, *L. casei*, *L. plantarum*, *L. rhamnosus*, *L. helveticus*, *L. gasseri*, *B. bifidum*, etc.), which opens promising avenues in the food industry [61]. Of note, the protection of these bacteria by chitosan could be one of the anti-obesity mechanisms.

## 5. Treatment of Obesity with Chitosan-Based Delivery Systems

Oral administration is the preferred and easiest route of drug administration for the long-term therapy of diseases and complications. However, the oral delivery of many therapeutic peptides and proteins is still an unsolved problem due to the size, hydrophilicity, and instability of these molecules [62]. Therefore, recent studies have focused on obtaining carriers for efficient drug administration in obese and diabetic patients. Due to the presence of amino groups, chitosan has a positive surface charge at slightly acidic pH. This charge can easily interact with the anionic mucin present in the mucosal layer. The mucoadhesive properties of chitosan promote its uptake by intestinal and nasal cells, and have become a promising basis for drug delivery. Chitosan can be used in various forms, and depending on the method of preparation, these can be nanoparticles, microspheres, capsules, hydrogels, conjugates, etc. The possibilities of chemical modification expand the applications of chitosan. Chitosan-based delivery systems have been shown to be biodegradable and have low toxicity in both in vitro and in vivo studies [63,64].

Chitosan was used as a matrix for pterostilbene (PS) drug delivery. PS was used in conjunction with hydroxypropyl β-cyclodextrin (HPβCD) to enhance its solubility. This complex was incorporated into chitosan nanoparticles (PS/HPβCD-NP). PS/HPβCD-NPs showed improved bioavailability compared to free PS. The nanoparticles were non-toxic, had an improved effect on obesity, reduced cholesterol levels, and helped to convert white fat (which stores fat) into brown fat (which burns calories). PS/HPβCD-NP was administrated orally to obese rats on an HFD diet. A significant anti-obesity effect was demonstrated, as evidenced by the histological examination, lipid profile, UCP1 gene expression, and protein levels of SIRT1, COX2, IL-6, and leptin [65].

Liang et al. delivered silymarin incorporated into hybrid nanoparticles (CS-LPN) of a lipid polymer containing chitosan (DD 75–85%, MW 50–190 kDa) to the livers of adiponutrin/patatin-like phospholipase-3 transgenic I148M mice, a model of non-alcoholic fatty liver disease (NAFLD). NAFLD is associated with insulin resistance and metabolic syndrome with excess lipid accumulation in liver tissue. Silymarin requires a delivery system as it has low bioavailability when taken orally due to poor aqueous solubility. HepG2 and Caco-2 cells had stronger uptake of CS-LPN nanocarriers treated with fat emulsion CS-LPN. In vivo experiments confirmed that CS-LPNs enhanced the efficacy of silymarin and suppressed lipid accumulation in the livers of mice. CS-LPNs effectively reduced blood lipid levels and decreased lipid accumulation. These results suggest that this delivery system may be used for the treatment of NAFLD [66].

Another drug, hesperidin, a flavanone glycosite, was delivered using chitosan cross-linked with thioglycolic acid (CT-TGA). CT-TGA acted as a physical barrier in the gastrointestinal tract and prevented the absorption of nutrients. No cytotoxicity or adverse immunological reactions in vivo were detected for CT and CT-TGA. Encapsulated hesperidin reduced the body weight of C57BL/6 mice by 41% compared to the group receiving HFD, and limited fat accumulation by inhibiting its absorption [51].

N-trimethylated chitosan (TMC) was used as a coating polymer to deliver celastrol (Cel), a bioactive component derived from *Tripterygium wilfordii* [67]. It is known that drug delivery using TMC coating has shown enhanced oral bioavailability [68]. Due to the protection provided by the TMC coating, Cel was stable in the gastrointestinal tract. In vivo tests showed that Cel-TMC significantly reduced body weight and blood lipid regulation, and suppressed fatty inflammation, WAT anti-angiogenesis, and WA T reduction [67].

Animal experiments show significant effects of chitosan supplementation; however, humans’ and rodents’ gastrointestinal tracts differ significantly as rodents are mostly herbivores, while humans are carnivores. Two recent meta-analyses collected clinical data up to the year 2019 on chitosan supplementation [69,70], which included more than 1500 persons. Both meta-analyses found statistically, but not clinically, significant differences in weight and body mass index (BMI) in the chitosan group versus the placebo. All studies included overweight persons with BMI>30. Two more recent clinical trials included 30 and 74 obese patients. Fatahi et al. demonstrated that chitosan supplementation significantly improved indicators of obesity (body weight: −3.6 kg, lipid triglycerides: −5.7, total cholesterol: −14, insulin: −5.5, adiponectin: 1.7 ng/dL, leptin −19.4, and neuropeptide Y–42 ng/dL). Valero-Pérez et al. used the chitosan preparation LipiGO, containing β-glucan and chitin–chitosan, a patented mix of natural polysaccharides from the *Saccharomyces cerevisiae* yeast cell wall. The subjects with obesity type 1 from the treated group experienced significantly reduced body weight (−5.3 kg) and BMI (−2) compared with those in the placebo group [71,72].

**Figure 1:**
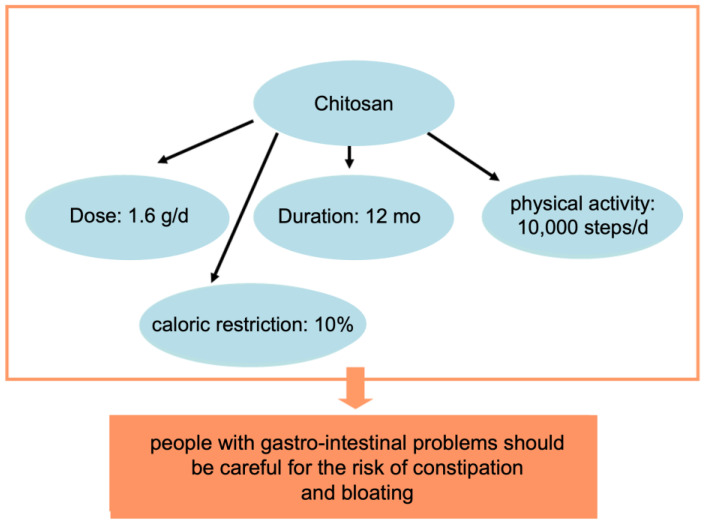
Loss of body weight during one year in kg.

Early studies used chitosan capsules for a month as a supplement to the normal diet, which resulted in the absence of an effect [73]. Later on, increasing the chitosan dose and duration to 2 mo resulted in a change of −1 kg in the chitosan group versus the placebo [74]. Another study, which tried chitosan treatment for 3 mo, already resulted in a change of −3 kg [75]. Finally, using a 1-year protocol supplemented with caloric restriction resulted in −10 kg of weight loss [76]. Taking these results collectively, it becomes clear that chitosan is not a magic anti-obesity drug per se; however, it presents an opportunity to lose some weight when it is used for longer, at a relatively high dose, in conjunction with a caloric restriction, and with increased physical activity (Figure 1). Currently, on the market, there are many different formulations of chitosan with anti-obesity activity and many more could appear (Table 4).

Chitosan can induce side effects at high doses, such as constipation and bloating, which can be resolved with increased water intake; however, people with gastrointestinal problems should be careful.

## 6. Conclusions and Future Directions

Obesity is a manifestation of metabolic syndrome, which has become a serious public health problem worldwide. Nowadays, there is a search for substances that could reduce the risk and manifestations of metabolic syndrome. Chitosan, a natural, biocompatible, biodegradable, and low-toxicity polymer may be suitable for this purpose. Summarizing the results, it can be concluded that all studied chitosan preparations (COS, medium- and high-MW chitosan, and their various derivatives and conjugates) are able to affect adipogenesis. To this end, average- to high-MW chitosans are cheaper than COS in preparation. Without not much difference in their activity, there is no need to produce more expensive COS. Empirically found commercial chitosan preparations have demonstrated good efficacy. We hypothesize that chitosan mostly acts as a flocculant, collecting charged molecules in the gut and promoting the chyme to move quicker in different parts of the digestive tract. The removal of extra chyme improves gut microbiota and intestinal barrier dysfunction and explains why all chitosan derivatives demonstrate comparable activity. This also explains why a prolonged diet is more effective than short courses. However, more studies are still needed to support or reject this hypothesis and further reveal the molecular mechanism of chitosan.

Multiple data show that chitosan supplements not only prevent obesity and dyslipidemia but also regulate BAT thermogenesis and hepatic lipid metabolism, protect pancreatic β cells, decrease insulin resistance, stimulate glucose uptake by muscle cells, improve the gut microbiota, and restore intestinal barrier dysfunction. This looks promising as chitosan supplements could affect/protect major tissues such as the pancreatic, adipose, intestinal, liver, and muscle tissues. To maximize the multi-targeted effects of chitosan preparations, oral application is recommended as the most effective pathway for its activity.

Thus, chitosan, a cheap and safe natural biopolymer, can be used as a safe food supplement that can help to prevent obesity and improve multiple body characteristics when used in a combination with a healthy diet and lifestyle.

## Figures and Tables

**Table 1:** Anti-obesity effects of COS.

MW, kDa	Cells, Species	Route	Doses, Duration	Anti-Obesity Effects	Ref.
1–10	3T3-L1	-	0.5–4 mg/mL	Decreased lipid accumulation, mRNA expression of C/EBP-α, PPAR-γ, leptin, adiponectin, and resistin levels	[26]
1–10	3T3-L1	-	0.6–4.8 mg/mL	Decreased lipid accumulation, free glycerol release, expression of adipogenicity marker genes, and activation of IL-6 and PTGS2 genes	[33]
5–10	3T3-L1	-	0.6–4.8 mg/mL	Suppression of lipid metabolism and lipid accumulation genes; inhibition of leptin expression	[25]
1–3	3T3-L1	-	0.5–5 mg/mL	Reduced level of intracellular lipid droplets and TG; decreased level of extracellular glucose	[34]
	Sprague–Dawley rats, HFD	Oral	600 mg/kg·day, 8 weeks	Significant reduction in weight gain and in TG, TC, LDL-C, glucose, and FFA levels; significant increase in HDL-C levels; effective suppression of adipose tissue hypertrophy and hyperplasia; and increased expression of UCP1, PGC1α, PRMD16, and ATF2 in white adipose tissue and brown adipose tissue	
≤1	Sprague–Dawley rats, HFD	Oral	300–1200 mg/kg·day, 8 weeks	Reduced body weight; suppressed perirenal, epididymal, subcutaneous, and total fat accumulation; reduced TC, TG, and LDL-C; and improved HDL-C levels in blood serum	[32]
≤1–3	Sprague–Dawley rats, HFD	Oral	150–600 mg/kg/day, 5 weeks	Mostly weight loss; significant decrease in serum total cholesterol and LDL-C levels; decrease in PPARγ and LXRα gene expression in white adipose tissue	[29]
≤1	Sprague–Dawley rats, HFD	Oral	150–600 mg/kg·day, 8 weeks	Inhibition of body weight gain; reduction in adipocyte hypertrophy and fat accumulation; reduction in hepatic steatosis; significant reduction in leptin; increase in LepRb expression and JAK2-STAT3 phosphorylation levels; inhibition of lipid synthesis in the liver; regulation of SREBP-1c, FAS, ACC, HMGCR, and adiponectin gene expression	[35]

HFD, high-fat diet; C/EBP-α, CCAAT/enhancer-binding protein-α; PPAR-γ, peroxisome proliferator-activated receptor protein γ; PG-endoperoxide synthase 2 (PTGS2); TG, triglyceride; LDL-C, low-density lipoprotein cholesterol; FFA, free fatty acids; HDL-C, high-density lipoprotein cholesterol; UCP1, uncoupling protein 1; PGC1α, PPARγ coactivator-1α; PRMD16, PR/SET Domain 16; ATF2, activating transcription factor 2; LXRα, Liver X receptor alpha; LepRb, long-form leptin receptor-b; JAK2-STAT3, Janus kinase-2-signal transducer and activators of transcription-3; SREBP-1c, sterol regulatory element-binding protein 1; FAS, fatty acid synthase; ACC, acetyl-CoA carboxylase; HMGCR, 3-hydroxy-3-methylglutaryl-CoA reductase.

**Table 2:** Anti-obesity effects of 20–740 kDa chitosan.

MW, kDa	Species	Route	Doses, Duration	Anti-Obesity Effects	Ref.
80	Sprague– Dawley rats, HFD	Food supplement	5%, 10 weeks	Suppression of weight gain; improved balance of plasma, liver, and feces lipids and intestinal disaccharidase activity. Reduced mucinase and β-glucuronidase activities in fecal microflora.	[44]
80, 740	Sprague– Dawley rats, HFD	Food supplement	5%, 8 weeks	Effective improvement of hypercholesterolemia and cholesterol homeostasis through activation and inhibition of hepatic AMPKα, PPARα, and intestinal ACAT2.	[38]
-	Sprague– Dawley rats, HFD	Oral	600 mg/kg·day, 8 weeks	Significantly reduced weight gain, TG, TC, and LDL-C levels, and glucose and FFA levels. Promotion of energy release; increase in HDL-C levels; suppression of adipose tissue hypertrophy and hyperplasia; and increased UCP1, PGC1α, PRMD16, and ATF2 in white and brown adipose tissue.	[34]
21, 46, 130	C57BL/6J mice, HFD	Oral	100 and 300 mg/kg·day, 10 weeks	Suppression of body weight and adipose tissue gain; induction of lipid-lowering effects; increase in fecal excretion of fat and/or bile acids; decreased absorption of triacylglycerol and cholesterol.	[43]
300	ICR mice	Oral	60 mg/kg·day, 5 weeks	Body weight reduction; epididymal fat pad and intra-abdominal fat thickness reduction; decrease in TC, TG, and LDL-C plasma levels.	[31]
-	Pigs, basal diet	Food supplement	300–1200 ppm, 1 week	Decreased crude fat digestibility, daily food intake, and final body weight; increased leptin concentration; and decreased serum C-reactive protein concentration.	[45]

HFD, high-fat diet; AMPKα, AMP-activated protein kinase α; PPARα, peroxisome proliferator-activated receptor protein γ; ACAT2, acetyl-coenzyme A acetyltransferase 2; TG, triglyceride; TC, total cholesterol; LDL-C, low-density lipoprotein cholesterol; FFA, free fatty acids; HDL-C, high-density lipoprotein cholesterol; UCP1, uncoupling protein 1; PGC1α, PPARγ coactivator-1α; PRMD16, PR/SET Domain 16; ATF2, activating transcription factor 2.

**Table 3:** Anti-obesity effects of chitosan derivatives.

Objects	MW, DD/DS	Cells, Species	Routes	Doses, Duration	Anti-Obesity Effects	Ref.
(N,O)-sulfatedchitosan	<1kDa	3T3-L1	-	0.1–4 mg/mL	Reduced lipid and triglyceride accumulation; facilitated lipolysis and mRNA expression; and reduced PPAR-γ receptor and C/EBP-α protein levels	[28]
Phosphorylated glucosamine(PG1c)	-	3T3-L1	-	0.2 mg/mL	Reduction in lipid accumulation; dose-dependent suppression of PPAR-γ receptor and C/EBP-α protein activity; induction of preadipocyte factor 1 mRNA activation; suppression of fatty acid synthase, lipoprotein lipase, and leptin	[27]
O-carboxymethyl chitosan (O-CMCs), N-[(2-hydroxy-3-N,N-dimethylhexadecyl ammonium)propyl] chitosan chloride (N-CQCs)	50 kDa, DD 85%; O-CMCs DS 72% N-CQCs DS 21%	3T3-L1	-	0.1 mg/mL	Suppression of leptin and resistin mRNA expression; increased adiponectin and PPAR-γ mRNA expression	[50]
		Clean Wistar rats, HFD	Oral	100 mg/kg·day, 6 weeks	Reduction in plasma leptin, glucose, insulin, and total cholesterol levels	
Chitosan –thioglycolic acid	-	C57BL/6 mice, HFD	Oral	250 mg/kg day, 8 weeks	Significant reduction in weight gain and fat distribution	[51]

HFD, high-fat diet; PPAR-γ, peroxisome proliferator-activated receptor protein γ; C/EBP-α, CCAAT/enhancer-binding proteins-α.

**Table 4:** Patents of chitosan-based applications to combat obesity.

Patent №	Patent Title	Description	Ref.
CN110477398	Application of chitosan in preparation of anti-obesity food	This invention relates to the use of chitosan in the preparation of food products against obesity. In a food product with a high fat content, chitosan is added in an amount of 3–10% of the weight of the product.	[77]
EP3225239	Body weight control preparation based on chitosan and cellulose	Compositions for oral administration are proposed that contain chitosan, capable of binding fatty acids and cellulose, and capable of forming a gel. These compositions are intended to combat obesity and overweight.	[78]
KR1020110010018	Chitosan mixture containing complex additives for antioxidation	A chitosan mixture containing added vitamin C and organic acid is presented, designed to suppress fat accumulation and enhance the fight against obesity.	[79]
KR1020090119319	Anti-obesity composition containing chitosan oligosaccharide and deep-sea water with fewer side effects	An anti-obesity composition is presented, containing chitosan oligosaccharides and deep-sea water with chlorine, sulfate, and magnesium. Designed to inhibit fat accumulation and for use as an additive in functional foods.	[80]
KR1020060082351	Functional anti-obesity biohealth material containing low-molecular-weight chitosan as effective ingredient	Anti-obesity biomaterials consisting of low-molecular-weight chitosan are proposed, which improve physiological metabolism in obesity by reducing cell mass and abdominal fat size and improving serum lipid levels.	[81]
KR1020050055643	Dietary agent comprising chitosan microparticles useful in health-promoting food for treating obesity	A dietary preparation containing chitosan microparticles is proposed, which can be used as a therapeutic food for the treatment of obesity without causing the so-called “yo-yo” phenomenon.	[82]
KR1020030056753	Composition containing mycelium of Lentinus edodes and Agaricus blazei Murill and water-soluble chitosan for controlling obesity	A composition is presented that contains as the main components a mixture of Lentinus edodes mycelium, Agaricus blazei Murill mycelium, and water-soluble chitosan. This composition is used as a healthy food product to combat obesity and prevent cancer.	[83]
JP2002104975	Anorectic agent	It is proposed to add an anorectic agent containing low-molecular-weight chitosan or its derivative to healthy foods and drinks to prevent obesity.	[84]

## Data Availability

Not applicable.
